# Kinetics of Langerhans cell chimerism in the skin of dogs following 2 Gy TBI allogeneic hematopoietic stem cell transplantation

**DOI:** 10.1186/s12878-016-0050-z

**Published:** 2016-04-27

**Authors:** Sabrina Peters, Christian Junghanss, Anne Knueppel, Hugo Murua Escobar, Catrin Roolf, Gudrun Knuebel, Anett Sekora, Iris Lindner, Ludwig Jonas, Mathias Freund, Sandra Lange

**Affiliations:** Department of Hematology, Oncology, Palliative Medicine, Division of Medicine, University of Rostock, Ernst-Heydemann-Str. 6, 18057 Rostock, Germany; Institute of Legal Medicine, Division of Medicine, University of Rostock, St.-Georg-Str. 108, 18055 Rostock, Germany; Electron Microscopic Centre, Division of Medicine, University of Rostock, Strempelstr. 14, 18057 Rostock, Germany

**Keywords:** Langerhans cells, Dogs, Stem cell transplantation, Chimerism, Nonmyeloablative

## Abstract

**Background:**

Langerhans cells (LC) are bone marrow-derived cells in the skin. The LC donor/recipient chimerism is assumed to influence the incidence and severity of graft-versus-host disease (GVHD) after hematopoietic stem cell transplantation (HSCT). In nonmyeloablative (NM) HSCT the appearance of acute GVHD is delayed when compared with myeloablative conditioning. Therefore, we examined the development of LC chimerism in a NM canine HSCT model.

**Methods:**

2 Gy conditioned dogs received bone marrow from dog leukocyte antigen identical littermates. Skin biopsies were obtained pre- and post-transplant. LC isolation was performed by immunomagnetic separation and chimerism analysis by PCR analyzing variable-number-of-tandem-repeat markers with subsequent capillary electrophoresis.

**Results:**

All dogs engrafted. Compared to peripheral blood chimerism the development of LC chimerism was delayed (earliest at day +56). None of the dogs achieved complete donor LC chimerism, although two dogs manifested a 100 % donor chimerism in peripheral blood at days +91 and +77. Of interest, one dog remained LC chimeric despite loss of donor chimerism in the peripheral blood cells.

**Conclusion:**

Our study indicates that LC donor chimerism correlates with chimerism development in the peripheral blood but occurs delayed following NM-HSCT.

## Background

Haematopoietic stem cell transplantation (HSCT) is an essential option for therapeutic treatment of malignant haematopoietic diseases. Nonmyeloablative (NM) HSCT is characterized by reduced intensity and toxicity [1, 2] and is therefore a treatment option for patients with contraindications (e.g. old age) who are not eligible candidates for conventional myeloablative (M)-HSCT [3]. The success of NM-HSCT in donor engraftment is (yet) associated with acute graft-versus-host disease (GVHD) rates affecting up to 50 % of the patients causing post therapeutic morbidity, mortality and decrease in quality of life [1, 4]. Acute GVHD typically develops within the first 3 months after M-HSCT and mainly affects the skin, but also the liver and the gastrointestinal tract [5]. Following NM-HSCT the signs and symptoms of acute GVHD are usually delayed and arise beyond day +100 [6].

Langerhans cells (LC) are CD1a positive bone marrow-derived dendritic cells located in the epidermis and mucous membrane [7, 8]. They are characterised by the presence of cytoplasmatic Birbeck granules [9]. LC are able to deliver antigenic information of their environment to the draining lymph nodes for presentation to the T lymphocytes [10]. In addition, LC might play an important role in skin GVHD [11, 12].

The origin of LC (donor or recipient) appears to be of importance in GVHD development [11, 13]. The engraftment kinetic of donor LC is influenced by the conditioning. In conventional M-HSCT the majority of LC are of donor origin as soon as day +40. After reduced intensity conditioning the engraftment of donor LC is delayed and full donor LC chimerism is not detected before day +100 [12]. However, data regarding LC kinetics after NM-HSCT are rare and the correlation between LC chimerism and development of GVHD remains to be investigated.

For preclinical studies, especially in the field of HSCT, the dog has proven as unique model organism for decades due to high transferability potential of the gained results to humans [2, 14]. Canines and humans show common similarities in physiology, metabolism and lifespan of blood cells [15]. The clinical application of NM-HSCT in humans is based on a meanwhile well-established canine NM-HSCT model using 2 Gy total body irradiation for conditioning [14].

Lowering the intensity of the conditioning appears to increase the incidence of graft rejection [16]. Therefore, the development of new NM-HSCT regimens, e.g. application of new immunosuppressive drugs, is required. Hence, our present study was initially designed to assess the impact of the new immunosuppressant everolimus in the canine NM-HSCT model. In general occurrence of GVHD in the canine matched-sibling NM-HSCT model is rare, and thus the herein used experimental setting is not suitable for methodical GVHD studies. However, the development of donor LC chimerism following NM-HSCT is an observed phenomenon providing an important issue in transplantation LC biology that can be adequately investigated with this model.

In this study we therefore described the kinetics of LC number and chimerism in a canine 2 Gy NM-HSCT model to give a first insight into the role of LC in NM-HSCT.

## Methods

### Laboratory animals

Experiments were approved by the regional review board of the state Mecklenburg-Vorpommern (State Institute for Agriculture, Food Safety and Fishery Mecklenburg-Vorpommern, Germany; AZ: 7221.3-1.2-039/06) under advice of the regional animal ethics committee (§15 committee). Litters of beagles were obtained from commercial kennels licensed by the German Department of Agriculture. All dogs were dewormed and immunized against rabies, parainfluenca, leptospirosis, distemper, hepatitis, and parvovirus. Dog leukocyte antigen (DLA)-identical donor/recipient sibling pairs were selected on the basis of matching for highly polymorphic DLA class I and class II microsatellite markers [17, 18].

### Haematopoietic stem cell transplantation

Animals were treated according to a protocol evaluating everolimus as new immunosuppressant in a NM-HSCT setting [19]. Briefly, dogs were conditioned at day -1 with 2 Gy total body irradiation and received unmodified bone marrow from DLA-identical littermates at day 0. Marrow grafts contained a median of 3.7 × 10^8^ (range 1.9–11.8 × 10^8^) total nucleated cells/kg, 6.7 × 10^6^ (2.6–18.2 × 10^6^) CD34+ cells/kg and a median of 2.0 × 10^7^ (range 0.9–7.9 × 10^7^) CD3^+^ cells/kg (Table 2). Immunosuppression consisted of cyclosporin A (15 mg/kg BID) from day -1 to +35 and everolimus (0.25 mg BID) from day 0 to +27.

### Preparation of Langerhans cells

Tissue samples of the skin were obtained from the neck of 9 dogs before and after HSCT on days +28, +56 and +105 under general anaesthesia (punch biopsies, 2 × 50.5 mm^2^). In long-term chimeras specimen of dermal tissue were also taken after day +105. Tissue samples were disinfected in povidone-iodine (Mundipharma, Limburg/Lahn, Germany), bleached with sodium thiosulfate (0.05 %, Sigma Aldrich, Hamburg, Germany) and washed in phosphate buffered saline (PBS, Biochrom AG, Berlin, Germany). The epidermis was separated from the dermis by digestion with dispase (2.24 U/ml, Roche, Mannheim, Germany) at 4 °C overnight and at 37 °C (water bath) for one additional hour. Subsequently the epidermis was incubated at 37 °C for 30 min in trypsin (0.25 %, Biochrom AG) with DNase (10 μl/ml, Roche) to obtain a single cell suspension.

Single cells were labelled with a monoclonal mouse anti-canine CD1a antibody (clone CA9.AG5; kindly provided by Dr. P.F. Moore, School of Veterinary Medicine, University of California). Afterwards cell suspension was incubated with a goat-anti-mouse MicroBead (Miltenyi Biotec, Bergisch Gladbach, Germany). The labelled LC were enriched by MiniMACS device using large cell columns (Miltenyi Biotec).

### Blood preparation and chimerism analyses

Before and after HSCT peripheral blood of the recipients was taken weekly up to day +77 and in larger intervals thereafter for analyses of the donor/recipient haematopoietic chimerism. Granulocytes and peripheral blood mononuclear cell (PBMC) fractions were separated by standard Ficoll-Hypaque density gradient centrifugation (density 1.074 g/ml).

Genomic DNA of LC was isolated using *Genomic DNA from Tissue-Kit* (Macherey-Nagel, Düren, Germany). Genomic DNA of granulocytes and PBMC was isolated using *Nucleobond CB 100-Kit* (Macherey-Nagel). Subsequently, polymorphic tetranucleotide repeats were amplified by PCR using commercially fluorescein-labelled primers (BioTez Berlin-Buch GmbH, Berlin, Germany) according to standard protocols. PCR-products were analysed by capillary electrophoresis as described elsewhere [20].

### Statistics

The Mann-Whitney *U*-Test was performed to compare LC cell counts between dogs that rejected the graft and long-term chimeras. Data of LC chimerism versus chimerism in the peripheral blood were analysed by the Wilcoxon test. Correlations between LC chimerism and chimerism in the peripheral blood compartments were evaluated using the Spearman’s rank correlation coefficient. Probability of *p* < 0.05 was considered significant.

## Results

### Cell purity and yield

**Fig. 1:**
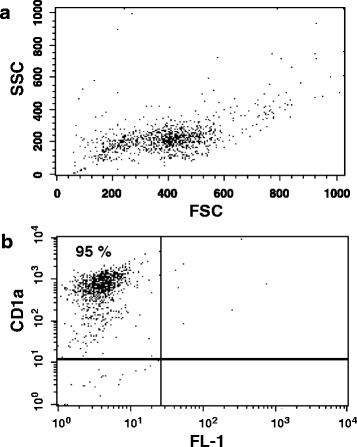
Representative flow cytometric analysis of CD1a expressing epidermal canine Langerhans cells (LC). **a** Forward scatter (FSC) and side scatter (SSC) characteristics of canine LC. **b** FACS dot plot showing a purity of 95 % CD1a expressing LC after isolation with MiniMACS technology

Punch biopsies of the skin from 9 dogs were obtained before and on days +28, +56 and +105 after NM-HSCT. Flow cytometric analyses of isolated LC revealed a purity of CD1a positive cells of median 91 % (range 28–97 %) (Fig. 1). Absolute LC cell counts showing a median of 3.0 × 10^4^ (range 0.8–13.5 × 10^4^) per 100 mm^2^ biopsy were obtained before HSCT. After transplantation a decrease in LC to a median of 1.5 × 10^4^ (range 0.3–5.6 × 10^4^) was detected at day +28. Normal counts of 3.0 × 10^4^ could be reached at day +56 after HSCT (Table 1). Differences in LC counts between dogs that rejected the graft and long-term chimeras were not observed.

**Table 1:** Langerhans cell counts and purity following isolation and enrichment

	before HSCT		d +28		d +56		d +104-112		d +140-280		d > +280	
	cells [x10^4^]^a^	purity [%]^b^	cells [x10^4^]	purity [%]	cells [x10^4^]	purity [%]	cells [x10^4^]	purity [%]	cells [x10^4^]	purity [%]	cells [x10^4^]	purity [%]
No. 1	2.3	69.4	1.5	81.2	0.8	82.2	0.4	80.5	n.d.	n.d.	n.d.	n.d.
No. 2^c^	1.9	93.2	0.8	79.2	3.8	87.5	^c^		^c^		^c^	
No. 3	2.0	41.5	0.3	69.0	1.5	68.6	1.9	68.2	4.1	84.1	6.4	92.7
No. 4	0.8	82.4	1.5	73.2	1.1	71.9	4.3	78.4	7.1	88.7	10.5	94.3
No. 5	13.5	92.0	5.6	89.8	11.6	92.7	9.7	96.7	n.d.	n.d.	9.0	94.2
No. 6	3.0	27.7	2.0	34.0	3.0	66.8	1.5	67.0	1.1	86.7	6.4	93.2
No. 7	4.5	96.7	3.4	72.9	7.2	87.5	0.3	78.1	4.5	93.3	4.1	92.2
No. 8	5.3	97.3	0.8	79.1	5.3	90.9	6.0	87.5	8.6	92.1	1.9	87.2
No. 9	4.1	91.2	1.9	87.2	2.6	93.5	3.4	89.2	6.0	90.0	n.d.	n.d.
Median	3.0	91.2	1.5	79.1	3.0	87.5	2.7	79.5	5.3	89.4	6.4	93.0

^a^cell counts per 100 mm^2^; ^b^purity = % of CD1a + cells; ^c^died day +60; n.d. not determined

**Fig. 2:**
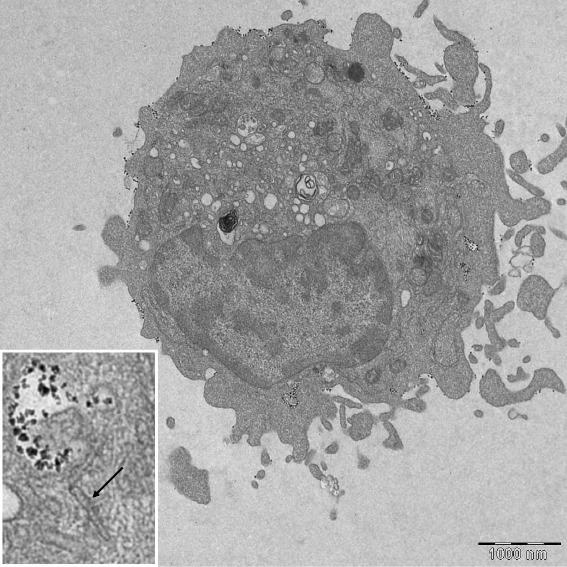
Electron microscopic image of a Langerhans cell. The figure insert shows a characteristic Birbeck granule (*black arrow*)

To verify that the enriched CD1a positive cells were true LC, electron microscopic identification of LC-characteristic Birbeck granules were performed (Fig. 2).

### Chimerism

All dogs initially engrafted (Table 2). Three dogs (No. 1, 8, 9) rejected their grafts before day +100 (days +70, +70, +91). Dog no. 6 had a late rejection more than 1 year after HSCT (day +391). One animal (No. 2) died at day +60 due to an infection. At day +28 the donor chimerisms in the granulocyte and PBMC compartments were median 55 % (24–100 %) and 26 % (14–58 %) in all dogs, respectively. In none of the animals LC donor chimerism could be demonstrated at that time.

**Table 2:** Graft composition and donor percentages of mononuclear cells of the peripheral blood (PBMC) and Langerhans cells (LC) after transplantation

Dog	Graft composition			Donor chimerism [%]										
				d +28		d +56		d +104-112		d +140-280		d > +280		rejection
	TNC [x10^8^]	CD34 [x10^6^]	CD3 [x10^7^]	PBMC	LC	PBMC	LC	PBMC	LC	PBMC	LC	PBMC	LC	(day)
No. 1	2.7	4.0	0.9	26.3	0.0	7.7	0.0	0.0	0.0	n.d.	n.d.	n.d.	n.d.	+70
No. 2	6.4	13.7	3.7	41.0	0.0	36.4	1.9	^b^		^b^		^b^		died d + 60
No. 3	3.7	8.2	2.0	31.0	0.0	67.4	6.3	100.0	16.5	100.0	55.4	100.0	88.6	no
No. 4	1.9	3.6	1.8	17.8	0.0	64.1	41.9	100.0	88.5	100.0	90.0	100.0	95.6	no
No. 5	11.8	10.3	7.9	31.7	2.0^a^	49.7	16.3	63.9	13.9	n.d.	n.d.	80.1	81.4	no
No. 6	6.2	18.2	3.4	58.1	0.0	39.9	0.0	41.1	12.1	7.7	17.0	0.0	35.9	+391
No. 7	7.6	6.7	3.5	25.9	0.0	21.1	2.3	92.3	3.8	25.4	6.2	17.5	22.7	no
No. 8	2.2	2.6	1.1	14.7	7.2^a^	3.1	7.2	5.0	5.1	0.0	7.9	0.0	5.0	+70
No. 9	3.1	3.0	1.8	14.0	0.0	10.5	0.0	0.0	0.0	0.0	0.0	n.d.	n.d.	+91
Median	3.7	6.7	2.0	26.3	0.0	36.4	2.3	52.5	8.6	16.6	12.5	48.8	58.7	

n.d. not determined

^a^LC chimerism test results for these dogs were already 3 % (No. 5) and 7 % (No. 8) before transplantation despite repeated testing. Therefore, d + 28 chimerism might be considered as not present

^b^died day +60

**Fig. 3:**
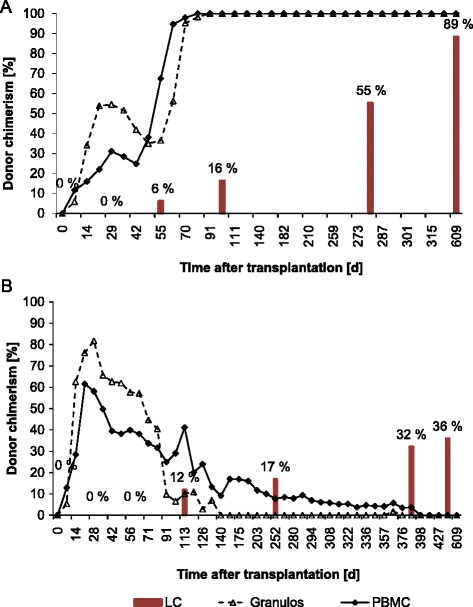
Chimerism kinetics of Langerhans cells (LC) in comparison to the peripheral blood chimerism. Development of LC donor chimerism (bars) compared with donor chimerism of PBMC (solid line) and granulocytes (dotted line) after 2 Gy nonmyeloablative HSCT in two dogs. **a** Dog No. 3 with full donor chimerism in peripheral blood. Continuously increasing LC donor chimerism starting at day +56 after HSCT at a time when the dog experienced strong engraftment in the peripheral blood. Donor chimerism of LC developed delayed compared to donor chimerism in the peripheral blood and did not achieve the peripheral blood levels during the observation period **b** Dog No 6 with initial engraftment and subsequent late graft rejection. Despite high initial donor chimerism levels in the peripheral blood of 82 % (granulocytes d +28) and 62 % (PBMC d +21) first LC donor chimerism was not detected before day +112 probably as a consequence of decreasing peripheral blood chimerism levels starting 4 weeks after HSCT. Interestingly, although donor chimerism values of the peripheral blood continuously declined and the graft was eventually rejected at day +391 a continuously increasing LC donor chimerism was observed also beyond the date of graft rejection

First LC donor chimerism was detected by day +56 in the dogs (No. 3, 4, 5, 7) that experienced a stable long-term chimerism in the granulocytes and PBMC compartments as well as in the dog that died. The median LC donor percentage of these five animals amounted to 6 % (2–42 %) at that time. Subsequently, a gradual increase in donor LC chimerism over the time was observed (exemplified by dog No.3 in Fig. 3a). The two dogs (No. 3, 4) that developed a full donor chimerism in the peripheral blood by days +77 and +91 also achieved the highest level of donor LC chimerism. Dog No. 4 showed the most rapid increase in donor LC percentage, and suffered as the only one from acute GVHD starting by day +70.

The dog (No. 6) that experienced late rejection showed first detectable LC chimerism not until day +112 although a donor chimerism in granulocytes and PBMC of 58 % and 40 % was already present at day +56. Interestingly, despite a subsequent decline in donor chimerism in the peripheral blood to 0 %, a constantly increasing LC donor chimerism up to 36 % (day +469) was observed (Fig. 3b). In contrast, in the dogs that rejected their grafts before day +100 LC of donor type could not be detected during the complete observation period.

In summary, donor LC chimerism was significantly lower than donor chimerism in the PBMC or granulocytes compartments (day +56: *p* = 0.011 each). Furthermore, there was a strong correlation between the PBMC donor chimerism and the donor chimerism in LC (day +56: *r* = 0.7, *p* = 0.038). Dogs that showed PBMC donor chimerism < 11 % at day +56 experienced early graft rejection and had no donor-derived LC at any time point. However, PBMC donor chimerism of 20–40 % at day +56 resulted subsequently in increasing LC donor chimerism despite decreasing PBMC chimerism. Only PBMC donor chimerism ≥ 50 % at day +56 correlated to high-level long-term engraftment in the peripheral blood and in the LC.

## Discussion

The aim of this study was to characterize the development of LC donor chimerism in the skin after NM-HSCT. For this purpose skin biopsies were taken from 9 transplanted dogs before and at different times after NM-HSCT.

Studies describing the kinetic of LC chimerism after myeloablative or reduced-intensity conditioning were conducted previously [12, 13], but data analysing LC chimerism following NM-HSCT still remain rare.

The herein gained results showed a moderate reduction of LC counts following NM-conditioning. The cell number decreased from day 0 to +28 by half (1.5 × 10^4^/100 mm^2^) and recovered to the initial value at day +56. In myeloablative regimens a LC nadir of 0.2 × 10^4^/100 mm^2^ during the first month was observed and the LC count increased to its normal level within 4-12 months after HSCT [21, 22]. These results demonstrated a considerably lower decrease and a faster recovery of LC numbers after NM-HSCT when compared to the kinetics seen in myeloablative regimens.

The donor LC chimerism following NM-HSCT increased slowly. In none of the examined dogs donor LC were detectable until day +56. Even at day +105 the present LC were mainly of host origin and the development of LC chimerism was not finished within 1 year after HSCT. In contrast, data from myeloablative studies certainly had shown a rapid replacement of host LC by donor derived LC as early as day +56 after HSCT [13]. The retardation in donor LC engraftment in our NM-HSCT study was even more pronounced than the delay previously reported after reduced-intensity conditioning, where by day +100 the majority of LC were donor in origin [12]. Previous studies demonstrated that the recruitment of circulating LC precursors does not only depend on proinflammatory chemokines as CCL20, but also on available LC sites in the epidermis [11, 23]. We assume that the availability of LC sites in the epidermis was reduced due to a less efficient depletion of host LC by NM-conditioning. Therefore, the recruitment of donor LC precursor could be hampered, beeing the reason for a delayed donor LC engraftment after NM-HSCT compared to myeloablative regimens.

In addition, a small fraction of LC is able to perform in situ proliferation [23–25]. This self-reproducing capacity may explain why the reduction of LC number by half was not followed by a 50 % LC donor chimerism after reaching initial cell counts in our study.

We also analysed the development of donor chimerism in LC comparatively to the ratio seen in granulocytes and PBMC. The significantly delayed donor LC engraftment in our dogs is in accordance with the reduced LC chimerism compared to DC chimerism in peripheral blood or the bone marrow as described in a current NM-HSCT study [26]. Dog No. 6 which experienced late graft rejection even displayed a continuous increase of LC donor chimerism, whereas chimerism in peripheral blood was not detectable any longer. This observation is potentially caused by the ability of LC to proliferate in the epidermis [23–25]. Furthermore, dogs showing a 100 % donor chimerism in granulocytes and PBMC also reached the highest LC donor chimerism. Correlation analysis confirmed a strong relationship between LC and PBMC chimerism in our study. In contrast, in previous publications no correlation between dendritic cell chimerism in the blood and in the skin has been described [11, 12].

One dog in this study suffered from acute GVHD after transplantation. The GVHD occurred at day +70 and the dog rapidly developed a high LC donor chimerism until day +105. Whether the earlier onset of donor LC chimerism has triggered GVHD, or whether the development of acute GVHD may have facilitated the rapid replacement of host LC with donor derived LC cannot be concluded from this single case.

## Conclusions

Our study indicates that LC chimerism kinetics are delayed following NM-HSCT compared to chimerism development in the peripheral blood. Highest donor LC engraftment rates were observed in dogs with full donor peripheral blood chimerism and the LC chimerism correlates with the chimerism in PBMC. The kinetic of LC chimerism after NM-HSCT seems to be delayed in comparison to published data on the development of LC chimerism after myeloablative and reduced-intensity conditioning as well. Recipient LC are present in the skin even 1 year after NM-HSCT. Whether this difference in the kinetic of LC chimerism might be responsible for the delayed onset of acute skin GVHD following NM-HSCT remains to be investigated in future studies.

## References

[1] Slavin S, Nagler A, Naparstek E, Kapelushnik Y, Aker M, Cividalli G (1998). Nonmyeloablative stem cell transplantation and cell therapy as an alternative to conventional bone marrow transplantation with lethal cytoreduction for the treatment of malignant and nonmalignant hematologic diseases. Blood.

[2] McSweeney PA, Niederwieser D, Shizuru JA, Sandmaier BM, Molina AJ, Maloney DG (2001). Hematopoietic cell transplantation in older patients with hematologic malignancies: replacing high-dose cytotoxic therapy with graft-versus-tumor effects. Blood.

[3] Diaconescu R, Storb R (2005). Allogeneic hematopoietic cell transplantation: from experimental biology to clinical care. J Cancer Res Clin Oncol.

[4] Khouri IF, Keating M, Körbling M, Przepiorka D, Anderlini P, O’Brien S (1998). Transplant-lite: induction of graft-versus-malignancy using fludarabine-based nonablative chemotherapy and allogeneic blood progenitor-cell transplantation as treatment for lymphoid malignancies. J Clin Oncol.

[5] Sung AD, Chao NJ (2013). Concise review: acute graft-versus-host disease: immunobiology, prevention, and treatment. Stem Cells Transl Med.

[6] Mielcarek M, Martin PJ, Leisenring W, Flowers ME, Maloney DG, Sandmaier BM (2003). Graft-versus-host disease after nonmyeloablative versus conventional hematopoietic stem cell transplantation. Blood.

[7] Banchereau J, Briere F, Caux C, Davoust J, Lebecque S, Liu YJ (2000). Immunobiology of dendritic cells. Annu Rev Immunol.

[8] Stingl G, Tamaki K, Katz SI (1980). Origin and function of epidermal Langerhans cells. Immunol Rev.

[9] Romani N, Clausen BE, Stoitzner P (2010). Langerhans cells and more: langerin-expressing dendritic cell subsets in the skin. Immunol Rev.

[10] Romani N, Brunner PM, Stingl G (2012). Changing views of the role of Langerhans cells. J Invest Dermatol.

[11] Merad M, Hoffmann P, Ranheim E, Slaymaker S, Manz MG, Lira SA (2004). Depletion of host Langerhans cells before transplantation of donor alloreactive T cells prevents skin graft-versus-host disease. Nat Med.

[12] Collin MP, Hart DNJ, Jackson GH, Cook G, Cavet J, Mackinnon S (2006). The fate of human Langerhans cells in hematopoietic stem cell transplantation. J Exp Med.

[13] Auffermann-Gretzinger S, Eger L, Bornhäuser M, Schäkel K, Oelschlaegel U, Schaich M (2006). Fast appearance of donor dendritic cells in human skin: dynamics of skin and blood dendritic cells after allogeneic hematopoietic cell transplantation. Transplantation.

[14] Storb R, Yu C, Wagner JL, Deeg HJ, Nash RA, Kiem HP (1997). Stable mixed hematopoietic chimerism in DLA-identical littermate dogs given sublethal total body irradiation before and pharmacological immunosuppression after marrow transplantation. Blood.

[15] Mack GS (2005). Cancer researchers usher in dog days of medicine. Nat Med.

[16] Baron F, Sandmaier BM (2006). Chimerism and outcomes after allogeneic hematopoietic cell transplantation following nonmyeloablative conditioning. Leukemia.

[17] Burnett RC, Francisco LV, DeRose SA, Storb R, Ostrander EA (1995). Identification and characterization of a highly polymorphic microsatellite marker within the canine MHC Class I region. Mamm Genome.

[18] Wagner JL, Burnett RC, DeRose SA, Francisco LV, Storb R, Ostrander EA (1996). Histocompatibility testing of dog families with highly microsatellite markers. Transplantation.

[19] Junghanss C, Rathsack S, Wacke R, Weirich V, Vogel H, Drewelow B (2012). Everolimus in combination with cyclosporin a as pre- and posttransplantation immunosuppressive therapy in nonmyeloablative allogeneic hematopoietic stem cell transplantation. Biol Blood Marrow Transplant.

[20] Hilgendorf I, Weirich V, Zeng L, Koppitz E, Wegener R, Freund (2005). Canine haematopoietic chimerism analyses by semiquantitative fluorescence detection of variable number of tandem repeat polymorphism. Vet Res Commun.

[21] Volc-Platzer B, Rappersberger K, Mosberger I, Hinterberger W, Emminger-Schmidmeier W, Radaszkiewicz T (1988). Sequential immunohistologic analysis of the skin following allogeneic bone marrow transplantation. J Invest Dermatol.

[22] Perreault C, Pelletier M, Landry D, Gyger M (1984). Study of Langerhans cells after allogeneic bone marrow transplantation. Blood.

[23] Merad M, Manz MG, Karsunky H, Wagers A, Peters W, Charo I (2002). Langerhans cells renew in the skin throughout life under steady-state conditions. Nat Immunol.

[24] Kanitakis J, Morelon E, Petruzzo P, Badet L, Dubernard J-M (2011). Self-renewal capacity of human epidermal Langerhans cells: observations made on a composite tissue allograft. Exp Dermatol.

[25] Czernielewski JM, Demarchez M (1987). Further evidence for the self-reproducing capacity of Langerhans cells in human skin. J Invest Dermatol.

[26] Mielcarek M, Kirkorian AY, Hackman RC, Price J, Storer BE, Wood BL (2014). Langerhans cell homeostasis and turnover after nonmyeloablative and myeloablative allogeneic hematopoietic cell transplantation. Transplantation.

